# Impact of COVID-19 on digital medical education: compatibility of digital teaching and examinations with integrity and ethical principles

**DOI:** 10.1007/s40979-021-00084-8

**Published:** 2021-09-07

**Authors:** Saskia Egarter, Anna Mutschler, Konstantin Brass

**Affiliations:** Institute for Communication and Assessment Research, Wieblinger Weg 92A, 69123 Heidelberg, Germany

**Keywords:** Medical education, E-assessment, E-learning, Digitalisation, COVID-19

## Abstract

The COVID-19 (coronavirus disease 2019) pandemic in 2020 has had a lasting impact on all areas of personal life. However, the political, economic, legal and healthcare system, as well as the education system have also experienced the effects. Universities had to face new challenges and requirements in teaching and examinations as quickly as possible in order to be able to guarantee high-quality education for their students.

This study aims to examine how the German-speaking medical faculties of the Umbrella Consortium of Assessment Network (UCAN) have dealt with the challenges but also the opportunities that the outbreak of the COVID-19 pandemic created in medical education and whether digitalisation has been driven forward as a result. In an initial online-survey we focused our questions on the current teaching situation with regard to digitised teaching content, the support or establishment of adequate framework conditions by the medical faculties and IT facilities and also the execution of examinations during the summer semester 2020.

Between August and September 2020, a total of 88 examiners, educators, dean of study and/or technical admins from 32 partner faculties took part in the survey. Students were not included in our survey. Most respondents stated that a switch to a digital semester had worked, the use of e-learning increased compared to previous semesters and that most courses could be converted, with the exception of practical courses, which were largely cancelled. The respondents also indicated that most examinations could still be taken, with the exception of practical examination formats, like Objective Structured Clinical Examinations (OSCEs). However, in the case of face-to-face examinations, strict distance and hygiene conditions had to be obeyed or there had to be a switch to distance-online examinations, which raised many open issues such as equal opportunities of students (technical equipment, internet access, premises) and attempts at deception (third-party help with the exam, mutual exchange between students, web search).

In conclusion, we identified several issues regarding the rapid transition to a digital semester due to COVID-19 which were categorised into the following topics: Face-to-face teaching could not take place, (2) know-how of educators, (3) integrity aspects, (4) technical aspects, (5) additional personnel required, (6) additional time and effort required for implementation of digital teaching. Our study shows that a switch to digital teaching and distance online examinations is feasible, but many problems were encountered concerning academic integrity and basic ethical principles still need to be solved. In order to investigate whether above mentioned issued could be solved one year after the transition to a digital semester, we conducted a second survey in which the 32 initially surveyed institutions were questioned again.

**Supplementary Information:**

The online version contains supplementary material available at 10.1007/s40979-021-00084-8.

## Introduction

Electronic learning (e-learning) and examinations (e-examination) are becoming increasingly popular in medical education (Amin et al. 2011; Choules 2007; Egarter et al. 2020; Kuhn et al. 2018) and an indirect survey showed that indeed two thirds of German universities already offer digital forms of learning (Schmid et al. 2016). This has also fundamentally changed the way teaching and learning is organised and altered the roles and requirement profiles of students, lecturers and faculty members (Kinshuk et al. 2016).

Medical schools were beginning to move from traditional forms of face-to-face lecture-based teaching to other modes that involve online, distance or e-learning. This slow transition to digital learning and teaching is due to the fact that numerous processes have to be implemented across the board at different levels of the educational institutions: strategic processes on the part of the management, cross-disciplinary processes at the competence centres of the educational institutions and professional processes of the teachers (Kuhn et al. 2019). In this context, it is also necessary to create a framework that suggests the legal requirements and does not violate ethical principles. Equal opportunities and privacy of students must also be respected. A deliberate transition to digital teaching is therefore necessary and was taking place in slow but progressive steps. The Corona Pandemic accelerated this slow concept in March 2020 and solutions had to be found quickly to offer digital teaching for medical students with various distance e-learning possibilities.

There are two different variants of e-learning. Firstly, the synchronous form, in which students and teachers are present at the same time, which ensures direct communication and interaction. And secondly, the asynchronous type, in which students can use previously deposited teaching materials or recorded lectures at any time (Lawn et al. 2017; Liu et al. 2016). The teaching materials are usually stored on learning management platforms, such as such as Moodle (Modular Object-Oriented Dynamic Learning Environment; Moodle 2020) or ILIAS (Integriertes Lern-, Informations- und Arbeitskooperations-System, German for “Integrated Learning, Information and Work Cooperation System”; ILIAS 2020), so that they are freely available to students. Especially the asynchronous e-learning has the advantage that the students are independent of time and place and thus have a certain flexibility to acquire the partly extensive teaching contents of medicine at their own learning pace (Daubenfeld et al. 2020). In this context, a systematic review by George et al. (2014) suggests that online e-learning is of equal value and possibly even better than traditional teaching methods.

Blended learning is characterised by the combination of traditional, synchronous face-to-face learning and asynchronous or synchronous e-learning (Dziuban et al. 2018; Kim et al. 2008). Thus, it represents a promising teaching alternative. In academia, this learning format has had a steady growth. The inverted classroom method (also known as “flipped classroom”, “flip teaching”, “flipped learning”, “inverted teaching”) is a blended learning method in which a self-learning phase (individual phase) is placed before a face-to-face phase (Tolks et al. 2016). The attendance phase should then be used to deepen and apply the knowledge acquired (Bergmann and Sams 2014). This method takes into account the changed behaviour of the users and offers teachers more freedom in the presence phase to design their lessons.

The COVID-19 global pandemic has resulted in unprecedented public health measures (O’Byrne et al. 2020). This has impacted the political, economic, legal and healthcare system worldwide and subsequently the education sector with many universities halting campus-based teaching and examinations, as the traditional teaching methods are not compatible with corona-related hygiene and social distancing rules (Alsoufi et al. 2020; Klasen et al. 2020; Mian and Khan 2020; Sandhu and Wolf 2020). For this reason, we postulate the assumption that there will be a change in teaching style towards digital teaching and aim to identify the impact of COVID-19 on the education in terms of teaching methods, examination procedures and various factors affecting values of academic integrity and ethics. Finally, we will discuss how the digital semester may impact medical education in the future and address briefly which ethical principles and integrity-related issues, such as equal opportunities, attempts at deception or maintaining privacy, still hinder a sustainable achievement of digital education in the future.

## Methods

### Study design and questionnaire development for the first survey

As part of the present study, a catalogue of questions was drawn up for a survey of German-speaking medical faculties of the Umbrella Consortium for Assessment Networks (UCAN). The purpose of this survey was to find out how the individual medical faculties have dealt with the challenges but also the opportunities that the outbreak of the corona pandemic created in medical education and whether digitalisation has been driven forward as a result. In a first step, a focus group (*n* = 4) was formed to facilitate well structured, clear and cohesive questions. The questions focused on the current teaching situation with regard to digitised teaching content, the support or establishment of adequate framework conditions by the medical faculties and IT facilities and also the execution of examinations during the summer semester 2020 (SS 2020), which lasted from 20th April 2020 until 1st November 2020.

The final version of the survey was organised into four sections and comprised 34 questions, including general information of the participants (six questions), questions regarding teaching within SS 2020 (seven questions), questions related to assessments in SS 2020 (15 questions), and finally overall impressions of the impact of the corona virus on medical education (six questions). Question types involved single choice (14 questions), multiple choice (“please select all that apply”; four questions), and free text answers (16 questions). Some questions were only asked after choosing distinct predefined answers. In addition, at the end of each section there was the possibility to make additive comments in free text form.

### Implementation of the first survey

All German-speaking UCAN partners (examiners, educators, dean of study and technical admins across 32 German, Austrian and Swiss medical and veterinary faculties) were contacted with an invitation to participate. The link to the questionnaire was sent out by e-mail on 14.08.2020. The end of participation was limited to 30.09.2020. A LimeSurvey was used to perform the online survey. The survey was conducted anonymously in German language exclusively (see Supplementary File 1 for the original survey). Supporting data and answers are available upon request from the corresponding author.

### Conducting the follow-up survey

To determine the extent to which the issues regarding the introduction of a digital semester identified by our first survey have been resolved one year later, a second shorter follow-up survey was conducted. First, a focus group (*n* = 3) was formed again to allow for well-structured, clear and coherent questions. The follow-up questions focused on the extent to which solutions to the problems that arose during the transition to a digital semester could be solved and the extent to which a continuation of digitalisation in education is envisaged after the corona pandemic has been contained. This time, demographic data was not retrieved. The survey was also divided into four sections and contained 18 questions, 11 of which were only visible to the participants with a certain pre-crossed answer. The three sections included (1) technical aspects regarding teaching and examinations (three questions), (2) digitalisation of teaching and examination (five questions) and (3) examinations (ten questions; eight only appear in case of performing distance-online-examinations). Only single choice (*n* = 7) and free text (*n* = 11) questions were used. The link to the LimeSurvey was sent to the same UCAN-partner institutions by e-mail on 02.06.2021. All volunteers had the opportunity to participate until 16.06.2021. The survey was conducted anonymously in German language exclusively (see Supplementary File 2 for the original survey). Supporting data and answers are available upon request from the corresponding author.

### Evaluation of questionnaire and data analysis

For the first survey free text comments were categorised by the best fitting topic and ranked according to frequency. The answers to the question “*What barriers were encountered at your institution in the summer semester 2020*” were divided into the following six categories: (1) Face-to-face teaching could not take place, (2) know-how of educators, (3) integrity aspects, (4) technical aspects, (5) additional personnel required, (6) additional time and effort required for implementation of digital teaching. The answers to the question “*Please describe to what extent your teaching was affected by the corona pandemic in the summer semester of 2020*” were clustered in four categories: (1) Switch to digital teaching formats / cancellation of face-to-face teaching, (2) impact of the examination format, (3) integrity-related impacts and (4) other changes.

The purpose of the follow-up survey was to determine to what an extend previously identified issues could be solved. For this reason, the survey participants were asked whether their institutions had encountered the respective issue. An analysis of these repeated questions from the first survey was not undertaken. Data analysis, tables and figures were created using a combination of Excel, Word and PowerPoint (Microsoft Office 2019, Redmond, WA, USA).

## Results

### Overall characteristics of respondents of the first survey

Of 88 persons from 32 different UCAN partner institutions contacted, 47 participants from 14 different locations completed the questionnaire, yielding a response rate of 53%. Among these participants, 74.47% (*n* = 35) belonged to the study programme of medicine, 17.02% (*n* = 8) to the study programme of veterinary medicine, 2.13% (*n* = 1) to the study programme of dentistry and 6.38% (*n* = 3) did not provide any information on their respective study programme. Of the respondents, 46.81% (*n* = 22) were lecturers or teaching coordinators, 19.15% (*n* = 9) were examiners or exam coordinators, 12.77% (*n* = 6) were both and 21.28% (*n* = 10) were administrators.

### Impact of the corona pandemic on teaching in the health care sector

**Fig. 1:**
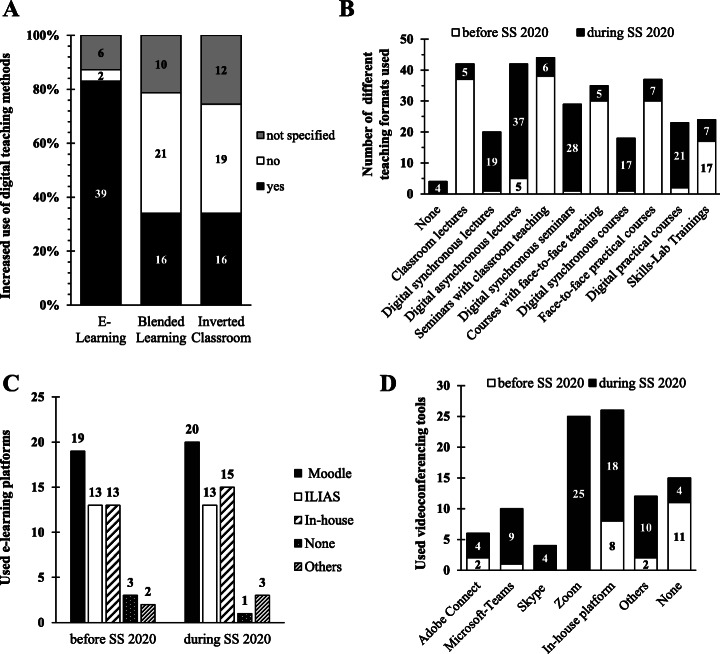
Change of teaching methods, e-learning platform and videoconferencing tool due to the digitalisation of medical education

Most respondents reported that there was still teaching provision at their institutions during the coronavirus pandemic. When questioned whether any teaching took place and which teaching formats were used in the SS 2020 compared with previous semesters, only four participants stated that no teaching courses took place. Almost 83% (*n* = 39) of the respondents reported an increase in the use of e-learning offers compared to previous semesters. In comparison, only 4% (*n* = 2) of the respondents stated that they had not increased their e-learning capacities. When asked whether blended learning or inverted classroom was used more often in the SS 2020, only one third of the respondents stated that either or both of the e-learning methods were more frequently used (Fig. 1A). Generally spoken most institutions managed to teach their planned lectures and seminars, practical courses, however, had to be suspended. Not surprisingly, face-to-face lectures, face-to-face seminars and face-to-face courses were converted into digital teaching formats. For lectures, asynchronous methods were used to a greater extent. While lectures and seminars could be digitised very well, only 44.7% of practical courses and 14.9% of skills-lab training could be conducted digitally (Fig. 1B).

Figure 1C shows the specific platforms, which were used by the participants for e-learning activities. No COVID-19-induced differences in the usage of e-learning platforms was identified. Only two participants stated that they had introduced a platform for the first time. The most frequently used e-learning platforms are Moodle, ILIAS and in-house platforms both before and during SS 2020.

Based on the increased use of digital teaching content, videoconferencing systems were also increasingly used. Almost 50% (*n* = 23) of the respondents did not specify on the usage of videoconferencing systems before SS 2020, 23% (*n* = 11) reported not having used a videoconferencing system and 27% (*n* = 13) were already using videoconferencing systems before the corona pandemic outbreak (Fig. 1D). In SS 2020 this scenario changed. Only 12.8% (*n* = 6) of the respondents did not specify and 34% (*n* = 16) use even more than one videoconferencing system with up to four different systems being used at the same location. The most widely-used systems for online teaching were Zoom, in-house platforms or Microsoft Teams (Fig. 1D).

### Execution of examinations in medical education during the corona pandemic

**Fig. 2:**
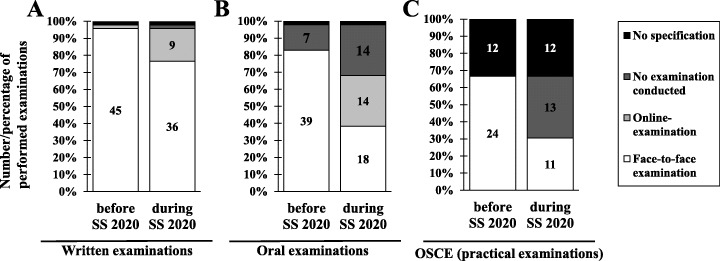
Execution of different types of examinations before and during Summer Semester 2020

Regarding the execution of examinations, 46/47 respondents indicated that examinations in general were still ongoing. However, only 61.7% (*n* = 29) of respondents had all examinations conducted, 29.8% (*n* = 14) explicitly stated they could not perform all examinations and 6.4% (*n* = 3) had to convert examinations into another format in order to conduct them. With regard to the evaluation of student performance, almost 50% (*n* = 23) declared that examinations that were not passed in the SS 2020 were not considered a failed attempt. In addition, one third of the respondents (*n* = 16) expressed that COVID-19 assignments by students were recognised as academic achievement at their respective institutions. Most respondents (except for one) stated that written examinations still could be conducted, and 20% (*n* = 11) declared a switch to online examinations instead of face-to-face exams (Fig. 2A). No remarkable difference regarding the used delivery method (paper, tablet, desktop-PC) of the exam was reported. The participants of the survey mainly used Moodle as platform for online exams, but also Microsoft Teams (Microsoft Teams 2020), Microsoft Forms (Microsoft Forms 2020) and the UCAN-ProgressTest (UCAN ProgressTest 2020) in combination with Zoom for proctoring was utilised. For oral examinations, a larger shift away from face-to-face examinations to either online exams (30%; *n* = 14) or no exam conduction (30%; n = 14) (Fig. 2B) was observed. Participants named primarily Microsoft Teams or Zoom as tool for conducting oral online examinations. The examination format with the most cancelled examinations is the practical format of Objective Structured Clinical Examinations (OSCEs). OSCEs are a widely established method for the assessment of clinical competencies including practical and communicative skills. 36/47 of the respondents used this format and 54% of those who specified their answer stated that they could not assess students using OSCEs in SS 2020 (Fig. 2C). No virtual performance of OSCEs was reported by the surveyed faculties and one respondent stated in the optional free text box “*OSCE examinations were assessed orally through practical stations*”.

### Barriers, limitations and future impact of digital education

In response to the open-ended free text question “*What barriers were encountered at your institution in the summer semester 2020*”, very diverse answers were given, which were retrospectively divided into six categories (Table 1). 22 participants stated that they had experienced technical problems at their institution. These problems included the lack of technical equipment or the failure/technical weakness of hardware or software. It was also noted that teachers often lack technical know-how or are not instructed properly. On the other side, some respondents stated that expensive equipment could no longer be used.

**Table 1:** Categorised answers to the question “*What barriers were encountered at your institution in the summer semester 2020*?”. Answers were retrospectively divided into the following six main categories: (1) Face-to-face teaching could not take place, (2) know-how of educators, (3) integrity-related aspects, (4) technical aspects, (5) additional personnel required, (6) additional time and effort required for implementation of digital teaching

**Technical aspects**	
Missing technical equipment	“*insufficient technical equipment”, “technical implementation”, “Insufficient supply of recording equipment”, “little or no equipment”, “lack of technical equipment, poor technical infrastructure”*
Failure of hardware or software	*“poorly functioning video conference software”, “Lack of server performance […*]”*, technical shortcomings of the ILIAS platform”, “technical difficulties, also on the part of the students”, “ILIAS platform was overloaded”, “Initially great difficulties with […*] *video conferencing tool”, “partly bad/very bad internet connection of institutions/offices/clinics”, “outdated technology”, “initially lack of software for digital teaching”, “no internet connections in classrooms, no recording technology, no transmission technology to larger lecture halls, […] poor intuitiveness of Moodle“*
Technical know how	*Inadequate instruction in digital technology”, “technical competence of the teacher”, “training of lecturers”, “Insufficient technical support”*
Unused technical equipment	*“tablet-based testing is not possible, expensively purchased equipment ultimately cannot be used for our subject in this semester, and is therefore superfluous”, “the pilot phase for tEXAM was postponed to the winter semester 2020/21”*
**Face-to-face teaching**	
No lectures, seminars, practical courses	*“Face-to-face events not possible or under very strict and elaborate hygiene conditions”, “Conversion of classroom teaching to digital teaching”, “practical courses were not possible in some cases”, “impossibility to offer practical exercises online”, “Furthermore, the practical implementation of internships, with the experience this entails for their future careers, can never be replaced by digital internships”*
No exams (e.g. OSCE)	*“large premises for* e.g. *face-to-face examinations are not available”, “large group examinations […] were or are very difficult to organise due to the regulations (distance rules), as suitable premises are lacking”, “all OSCE examinations were cancelled”, “A major organisational problem was the implementation of the face-to-face exams under the corona-related distance and hygiene regulations. Sufficiently large premises had to be found”*
**Additional personnel required**	
	*“A lot of work distributed among few people in the institution”, “personnel (no student assistant/secretary/team teaching”, “Not enough manpower”, “little or no manpower available for the creation of own educational films”, “Examination in small groups at 5 locations at the same time: requires many personnel resources”, “Unexpected considerable extra work through conversion to digital teaching”, “[…] and additional personnel were needed for supervision, disinfection and admission”, “Additional personnel required”*
**Additional time and effort**	
	*“teaching specifications by the faculty that are too tight in terms of time”, “Very short time window to convert the contents of the semester to completely digital”, “very limited time to implement digitisation”, “lack of time to build more complex courses in Moodle”, “No time to develop didactically more sophisticated concepts”, “little time for implementation”, “additional time and effort”, “material preparation strongly depends on individual willingness to invest additional hours (outside working hours!)”*
**Know-how of educators**	
Missing knowledge	*“untrained personnel”, “knowledge”, “no know-how on online teaching”*
Lack of willingness for digital teaching	*“Partial unwillingness of teachers to support digital teaching”, “[…] that digital teaching has been implemented very poorly or not at all, and they continue to “resist” the innovation”, “Reluctance to learn new things. Everyone wants to have some kind of service. Preferably personal support at every event”, “The challenge of adequately instructing all lecturers”, “in some cases little willingness to become acquainted with digital topics, analogue ideas are to be implemented 1:1 digitally”, “Reservations about digital teaching formats”, “Extremely good and fast support from the university with regular training (technology and didactics) for lecturers and students”*
**Integrity-related aspects**	
	*“few feel responsible”, “students’ complaints, lack of personal support”, “To find suitable alternatives for high-quality online teaching that was optimal for both teachers and students”, “high demands of students on digital teaching and its prompt implementation”, “Difficulties in obtaining royalty-free image, sound and video material”, “[…] solving the weekly quizzes to confirm participation”, “lack of uniformity (each subject had different platforms or different digital teaching methods)”, “support with regard to data protection; setting up a university video server with videoconferencing software for all video conferences, etc”, “Additional formalities for each procedure”, “On-site and face-to-face teaching and examinations continue to be very important, as this is the only way to get the best feedback on how students have problems, what they could understand well and what they could not”*

Nine of the respondents clearly stated that face-to-face teaching could no longer take place and that there was an impact on examinations. Eight of the respondents stated that the switch to a digital semester required additional personnel resources and seven participants answered that additional time and effort was required. Ten respondents indicated that obstacles arose due to a lack of know-how of educators. These barriers were broken down into lack of knowledge and lack of willingness for digital teaching. A further 11 respondents encountered obstacles that affected different aspects of academic integrity such as responsibility, dissatisfaction, ethical issues, legal formalities and compliance and creation standard procedures for conducting digital teaching and examinations, such as uniform software solutions.

In response to the free text question “*Please describe to what extent your teaching was affected by the corona pandemic in the summer semester of 2020*”, 42 of the respondents said that face-to-face teaching was cancelled at their institution and largely replaced by digital teaching. We further categorised these 42 answers into the following six sections: Switch to digital teaching formats (*n* = 29), cancellation of face-to-face teaching (*n* = 12), synchronous digital teaching methods used (*n* = 6), asynchronous digital teaching methods used (*n* = 8), new teaching material generated (*n* = 7), face-to-face teaching under hygiene regulations (*n* = 3). In addition, six participants indicated that there was an influence on the examination format. For some, face-to-face examinations were conducted online, while others split large cohorts of examinees into smaller cohorts spread over several examination rooms. Furthermore, seven respondents encountered integrity related barriers. These related to data protection conformity of digital teaching, legal requirements, but also to the lack of direct personal contact with students and, as a result, a lack of trust. One respondent specified that “*Question forums [were] established and processed*” to keep in contact with the students.

When asked about the future of digital conversion of teaching and examinations, 97.9% of participants (*n* = 45) consider the digitalisation of teaching and examinations to be important for the future. Furthermore, 76.6% (*n* = 36) think that the e-learning can partially replace “face-to face” education, while 19.1% (*n* = 9) stated no complete replacement will be possible. This opinion is even more pronounced for online examinations and 31.9% think that face-to-face exams cannot be substituted through distance online examinations. However, 53.2% of respondents said that partial replacement is possible. Overall, 93.6% (*n* = 44) of the respondents believe that the experience gained during the pandemic with regards to e-learning methods and online examinations will be used for future teaching and assessments respectively.

Fitting together with the future use of digital teaching and examinations, the following voluntary comments were made within the survey:

“*The pandemic has given a massive boost to the digitalisation of teaching - even if unprepared. Nevertheless, a sense of proportion is required for meaningfulness and didactics; “digital“ cannot be implemented equally in all subjects, but can bring new degrees of freedom into teaching”*; “*Online examinations where the examinee is not present? I see major legal difficulties here and do not believe that they will become established in the foreseeable future for important summative examinations*”; “*Lectures in face-to-face form are the least missed. A serious shortcoming was the absence of the preparation course and the resulting missing exchange* “; “*We continue to regard the digital media only as a (reasonable!) crutch until the return to the face-to-face teaching. Certain teaching contents (practical training!) are not possible digitally*”.

### Digital breakthrough after two digital semesters?

For our second survey, we contacted 85 educators, dean of studies and/or technical admins from 32 partner faculties of whom 19 completed the survey. During our first survey, it became clear that there were obstacles with regard to both teaching and examinations because adequate technical equipment was not available. Of the participants surveyed, nine stated that that they lacked appropriate equipment, seven had the necessary technical equipment and three made no statement. In response to the question “*What is the situation regarding technical equipment in the meantime? Have any sustainable solutions been created*?” it was stated that “*Various new acquisitions, but no comprehensive solution*” had been purchased and that “*the equipment has been standardised and increased in many places. Larger investments, e.g., to make lecture halls streaming-capable, could only be implemented in isolated cases due to the costs*”. The question of whether uniform, interdisciplinary structures could be created was answered “*no*” by only two participants. Seven said that there is a higher degree of standardisation and fewer individual solutions. Ten participants abstained. When asked if new staff resources could be created, nine respondents answered “*no*”, five respondents indicated that temporary positions were created and five did not specify. In our first survey, several participants stated that lecturers were not inclined to switch to digital teaching, lecturers did not have sufficient training in digital teaching, good didactic concepts were neglected in order to push ahead with digitisation quickly (the technically simplest solution was sought). Five respondents stated that training was offered at their faculties and that, overall, digital competence and the willingness of lecturers to teach digitally had improved. Regarding distance online examinations, we wanted to know whether the examination regulations have been adapted to allow this form of examination. While six participants stated that this was the case for them, 11 participants answered this question in the negative. Eight participants stated that at their university distance-online-exams are performed. Of the respondents whose faculties used distance-online exams, three indicated that a proctoring solution was used to monitor students. Only one respondent stated that there was evidence of cheating in the online exams. In this case, the students had communicated with each other. Two participants reported that there were rule violations, but that these did not lead to cheating. Regarding the average grades of distance online exams and face-to-face exams, no significant difference could be found overall (similar average grade, *n* = 3; slightly easier, *n* = 2 and minimally more difficult, *n* = 1). Three surveyed participants stated that the question formats were adapted for use in online examinations and/or the time of the examination was more limited, so that a quick “googling” of the answers or consultation with fellow students was made more difficult. Only half of the distance online examiners would like to continue using this examination format after the corona-related hygiene conditions have been abolished; the other half would like to switch back to face-to-face examinations at the universities.

## Discussion

### Opportunities and pitfalls within student-directed e-learning

Our survey shows that the COVID-19 induced short-term conversion to a purely digital education was associated with great difficulties in many places which is in line with other studies on this global issue (Al-Balas et al. 2020; Alkhowailed et al. 2020; Chatziralli et al. 2020; Mian and Khan 2020; O’Byrne et al. 2020; Tabatabai 2020). It would actually have been expected that in a world that has been digitalised in many areas, a rapid conversion to a virtual teaching environment should be relatively easy to master. Nevertheless, several unforeseen issues had to be overcome during the digital SS 2020. The evaluation of the free text comments in our survey revealed that through the use of asynchronous teaching methods, the direct contact between students and educators was missing and as a result the otherwise often familiar relationship between students and teachers, which builds up over time, could not develop properly. Non-verbal aspects of communication cannot be perceived and responded to as well or at all in a digital setting as in a direct face-to-face relationship (Bambaeeroo and Shokrpour 2017). Not only the teachers but also the students had to adapt to this new teaching method. The teaching content is now no longer taught centrally and synchronously, which can be an advantage since the students are now flexible regarding their own learning pace, but it also means that it is the responsibility of the students themselves to organise their individual learning units. The motivation for self-study is an aspect that should not be underestimated and is often characterised by the students’ postponement behaviour (Tillmann et al. 2016).

Although asynchronous teaching enables students to work independently at their own pace (independent of time and place), regular, personal contact between students and educators, as well as individual feedback, must still be guaranteed. In addition to content and method, students value reliable structure, organisation and support in the virtual environment. However, students need certain self-regulation strategies so that online courses are not accompanied by boredom and less enjoyment compared to face-to-face units (Stephan et al. 2019). Self-regulation can be promoted, for example, through portfolio work (Gläser-Zikuda et al. 2018). Asynchronous, digital blended learning/inverted classroom concepts can strengthen clinical teaching (Engel et al. 2019; Northey et al. 2015). Students work together on case studies and projects and apply their knowledge from the synchronous lecture in more complex contexts. The students’ motivation can be increased through the self-determined selection of topics. In interactive small groups, the solutions worked out can be presented and mutual feedback exchanged. The focus shifts from passive content transfer to interactive handling of content. Additionally, some faculties have introduced weekly quizzes to increase student motivation and also to check their “presence” on the relevant learning platforms. Virtual lectures can be designed to be more exciting through integration of small quizzes or surveys.

### Challenges of distance online examinations

The corona pandemic and the associated distance and hygiene regulations affected not only face-to-face teaching in medical education, but also the examinations required for assessing students’ performance. At many universities these could no longer be conducted in large cohorts. Since no generally applicable rules were established, very different individual solutions were found in the various faculties across Germany. While most surveyed institutions adhered to presence-based written examinations and divided large cohorts into small groups in several examination rooms or used large premises, such as canteens, for the examination of large cohorts, almost 20% (*n* = 11) of the respondents in this study switched to distance online examinations. Due to the uncertainty in dealing with the hygiene and distance regulations, the examination format was changed in many places. Some have moved away from electronic written examinations (tablet-based, desktop-based) back to paper. The reasons for this were relocation to non-computer-based premises or the risk of virus transmission during final cleaning of equipment after use. Others indicated that there had been a switch from paper-based examinations to electronic examinations. This change of medium could have been accompanied by a switch to distance online examinations. The implementation of distance online examinations is a challenging task and also requires faculties and universities to impose and develop a range of procedures, policies and activities to overcome unequal opportunities Both digital teaching and digital examinations can lead to the exclusion of students due to non-existent access to equal opportunities for digital or blended learning formats. It is difficult to ensure equal opportunities for all students, because the quality of technical equipment of the students is very diverse and thus advantages due to newer or better hardware or disadvantages due to poorer internet connection emerge. In order to overcome unequal opportunities, universities could provide technical equipment or the possibility to conduct online examinations on university premises, if necessary (Fuller et al. 2020). Inadequate technical equipment must also be taken into account in the legal certainty of examinations. What happens if a student with poor performance intentionally leaves the online examination and blames it on a lack of internet connection? Attempts at fraud are of similar importance. How can it be ensured that the correct student takes the examination and does not attempt to cheat? Certainly there is a wide range of technical software tools that are designed to prevent attempts at deception by means of so-called proctoring (Camara 2020; Guangul et al. 2020). Often it is handled in such a way that the students need two devices with cameras to transmit a picture of themselves at the workplace as well as of their surroundings. Of course, this immediately entails the legal risk that the students’ privacy is not respected. Furthermore, the use of such programmes can disturb the trust and relationship between teachers and students as it is already assumed in advance that fraud could take place (Mellar et al. 2018.).

On the other hand, however, such examinations must also stand up to legal certainty and also be represented in the examination regulations of the respective federal states. The changing nature of teaching and examinations due to the coronavirus pandemic requires a revision of academic standards and formalities adapted to this extraordinary situation, including the examination regulations of the respective federal states and institutions (Sandberger 2020). In Germany, there are regulations in this respect both at federal state level and at the level of the corresponding higher education institutions, but general, concrete rules for online examinations still need to be defined. Bavaria was one of the first of the federal states to amend the Bavarian Higher Education Act (For detailed information see section 63 of Bayerisches Hochschulgesetz, 2006) on 24th July 2020 including distance online examinations and was even valid retrospectively for the whole SS 2020. Also, the Higher Education Act of North Rhine-Westphalia (For detailed information see Higher Education Act of North Rhine-Westphalia 2020, p. 207-302) was revised and distance online examinations are now possible. The above-mentioned challenges, which occur in written distance online examinations, are easier to handle in oral online examinations. This could explain why a higher rate of online examinations was achieved in this examination format. In contrast to written examinations, these are held individually between students and examiners via video conferencing tool, so that an identity check can be easily carried out. Equal opportunities aspects also play a rather subordinate role, as the exchange of information is verbal.

### Digital teaching and testing of practical skills

Many of the competences required for the later practice of medicine, dentistry or veterinary medicine can be transferred well into a digital setting. The importance of these skills for decision-making within patient care is well summarised in the following statement by Guerrero-Dib et al. (2020):

“Promoting and experiencing academic integrity within the university context has a twofold purpose: to achieve the necessary learnings and skills to appropriately perform a specific profession and to develop an ethical perspective which leads to correct decision making.”

However, the digital conversion of practical skills poses a challenge for clinical practical skills and particularly at the beginning of the COVID-19 pandemic practical courses were discontinued (Fig. 2C) leading to an associated loss of essential skills for the later working life of physicians, dentists or veterinarians. Digitalisation of physical examinations on patients is simply not possible. These still have to take place in person, but can be carried out with the best infection prevention measures in place (Boursicot et al. 2020). However, there are also some practical clinical skills for which a well-thought-out digitalisation strategy can partly replace classroom teaching. One example is the skills that are acquired through bedside teaching. During bedside teaching, students can practice medical activities in direct contact with patients, such as taking a medical history, performing a physical examination or introducing a patient (Aldeen and Gisondi 2006; Kroenke et al. 1997). The contact with the patient can take place via a video conference, so that students have the opportunity to follow the examination at the patient’s bedside and, if possible, to get involved (Hofmann et al. 2020). Complete digitalisation of bedside teaching is a good substitute, but it can only replace direct contact with the patient to a limited extent (Pudritz 2021). The learning of interprofessional and communicative competences is an important and essential part of medical education (Bagnasco et al. 2014; Buring et al. 2009; Hean et al. 2012). Concepts involving simulations, telemedicine or virtual patients, have proven suitable for learning and testing these skills digitally (Abdelaziz et al. 2021).

According to our study, a digitalisation of practical assessments (OSCEs) was not feasible at surveyed faculties. Interestingly, the University of Heidelberg conducted the first virtual OSCE including simulated actors in SS 2020 as part of the Master of Medical Education (MME) course of studies. The virtual OSCE was administered via videoconferencing tool and designed (Cantone et al. 2019) to specifically assess medical interviewing and interprofessional competencies (Pante et al. 2020). Also other universities and institution changed to a digital OSCE format (Cantone et al. 2019; Craig et al. 2020; Hopwood et al. 2020; Lara et al. 2020; Lawrence et al. 2020). Within these virtual OSCEs, competencies such as history taking, knowledge of physical examination manoeuvres, problem solving, decision making and counselling could be assessed. Nevertheless, in all the proposed variants of a virtual OSCE, no physical examinations could be carried out on patients and thus these skills of the students cannot be assessed digitally and require face-to-face assessments.

### Recommendations for the conversion to a digital semester

#### No individual solutions - digital transition is a collaborative task

At the beginning of the COVID-19 pandemic, all educators faced the same challenge: The entire analogue teaching had to be converted to digital formats in the shortest possible time without a preparation phase. For many educators working exclusively in face-to-face teaching, however, this digital conversion was entirely new territory and digital competences had to be acquired to a great extent first. From our point of view, one of the most fundamental tips is to manage the digitalisation as a community together intra- as well as inter-institutionally. Avoiding individual solutions may sound banal at first, but it can make a huge difference, especially when a change is necessary at short notice. The digital transformation is a collaborative task and requires lasting cooperation and a constant exchange of knowledge. In this way, experience can be exchanged and future developments can be jointly considered. Digital teaching should not be understood as a temporary task, but as a permanent goal. Accordingly, central structures must be established on institutional level and be sustainably financed. As a first instance educators should contact the respective support facilities at their universities or faculties. Most institutions have central support structures established that can be contacted for technical and didactical questions. Additionally, contact and exchange with educators who are experienced in e-learning has also proven successful. For this purpose, digital possibilities such as forums, platforms, etc. have been created at various universities so that educators can inform themselves, exchange information and network. Students were also integrated into the process and supported educators in the transition to online teaching (Abler et al. 2020). de Jong et al. (2020) published 12 tips on how to integrate digital teaching units into one’s own teaching.

#### New didactical concepts over technically simplest solution

The unexpected introduction of online teaching in March 2020 was very spontaneous for educators, without a phase of preparation and careful selection of didactically suitable methods and techniques. For teaching content to be communicated as quickly as possible, many educators relied on the technically simplest solution, while didactic concepts were neglected, often unwittingly. Initially, video conferencing and online seminars were primarily used, while didactic diversity was lacking. However, the learning content and learning objectives should still be in the centre of attention and the focus should not be on the technology but on the content-related competences. Reflecting on the motivations for previously used face-to-face teaching methods to impart skills, competences and knowledge could provide conclusions on which virtual teaching units need to be revised in order to bring didactics to the fore by means of good blended learning alternatives. Akin to teaching also the why, who, when and what of assessments need to be rethought (Fuller et al. 2020). In our opinion, it is difficult to transfer several hours of face-to-face teaching one-to-one into an online format. In this case, it is advisable to divide the teaching unit into several shorter units in order to avoid rapid fatigue or reduced attention of the students. For further reading on didactic teaching methods, we recommend the literature review by Challa et al. on modern techniques on teaching and learning in medical education (Challa et al. 2021).

#### Recommendations for conducting digital assessments

Prior to the corona pandemic, examinations at German universities were only partially conducted electronically. At the beginning of the pandemic, many individual solutions for online exams were created which was also an outcome of our survey. In the support facilities of many institutions, the main focus was on questions of legal security (38%) and functioning technology (30%), but also on related topics such as acceptance and fairness of digital forms of examination (Dreyer 2020). Software-based monitoring of students within online examinations by means of proctoring is considered rather critical and problematic. In our follow-up survey, it became clear that cheating attempts by students in online exams could hardly be detected. Guerrero-Dib et al. (2020) state in their work that academic integrity is much more than avoiding dishonest practices and is essential in any teaching-learning process focussed on achieving the highest standards of excellence and learning. According to a survey by Reedy and colleagues, both students and staff believe that deterrents to cheating behaviour are proctoring, student beliefs, question design, exam duration and marking practices (Reedy et al. 2021). Given these statements the focus should be less on the assumption that cheating could take place and the use of a monitoring system in online exams and more on the trust in students that they do not intend to cheat. Vučković et al. (2020) showed in their study that most students are capable in identifying ethical misconduct which also includes cheating in examinations. But they also state that not all ethical issues are clear to students and universities should organise trainings to increase the awareness of ethical misconduct. So, a first step in minimising fraud attempts is good ethical education of the students. Secondly, the exam format and question types should be adapted specifically for online examinations. Probably the simplest way to prevent students from copying each other’s answers is to randomise the exam questions. On the one hand, the order of the questions could be randomised and on the other hand, different examination questions could be used. However, these would have to cover an analogous subject area and be comparable in terms of difficulty in order to ensure a homogeneous and fair examination for all students. For this reason, the future should focus more on new examination formats that make the use of proctoring software obsolete. One example is the Open Book approach (Sarkar et al. 2021; Zagury-Orly and Durning 2020). In the OpenBook examination approach, cognitively and thematically demanding examination tasks are made available to the students on a fixed date. Afterwards, a certain amount of time is set aside for processing. The use of analogue and digital aids is explicitly permitted. Such approaches are well suited, for example, to test transfer skills. In this case, however, the questions must be chosen accordingly and be directed less towards knowledge-based and more towards application-oriented task types, such as multiple-choice questions with case vignettes or media content. Pettit et al. (2021) did a worth reading literature review on virtual exams, which looked in depth at the challenges of online examination. They describe different possibilities to assess clinical skills in virtual exam and offer question design strategies to mitigate cheating behaviour (Cluskey Jr. et al. 2011; Pettit et al. 2021) One must bear in mind that the development and formulation of new question types is usually resource intensive, which is why it is unfortunately still a vision for the future at many universities.

### Outlook and conclusions

This study examined the influence of the corona pandemic on digitalisation in medical education in German-speaking countries. It can be said that, especially with regard to digital teaching, immense progress has been made and new, innovative teaching methods have found their way into a field in which e-learning has only been used sporadically despite advancing digitalisation (Handke 2015). However, the quick conversion to distance learning also presented many hurdles and, for example, technical problems had to be solved in many places (Table 1) (Veasuvalingam and Goodson 2020). Even though the switch to a digital semester was successful after some start-up difficulties, many educators and students are lacking more direct ways of communication usually occurring in face-to-face teaching units and thus the otherwise existing trust between educators and students. For this reason, a critical selection of the experience gained in this digital semester should be made in order to ensure future development of teaching with adequate digital teaching content. The future switch to partially digitally taught teaching content was also formulated by some of the survey participants to have both the benefits from distance teaching as well as face-to-face teaching. In conclusion, it can be said that even after two digital semesters, a didactically meaningful transfer to digital teaching methods has not yet been fully achieved and distance online examinations cannot be implemented at many universities. Nevertheless, the COVID-19 pandemic took the digitalisation of medical education a decisive step forward.

### Limitations of the study

There are some limitations to our study: We only questioned UCAN partners of German-speaking countries, which is leading to the circumstance that only health professions are regarded in our survey and the outcome could be different in other programmes of study. Due to the rather low number of responses, it was not possible to categorise the answers by respondent characteristics, such as location, state or type of UCAN partnership. Another limiting point is that we did not take into account the students’ point of view in this survey. This could still be done in the future and we might identify further aspects that are needed for the successful digitalisation of education in the healthcare system.

## Supplementary Information

**Additional file 1.**

**Additional file 2.**

## Data Availability

The datasets used and/or analysed during the current study are available from the corresponding author on reasonable request.

## Abbreviations

COVID-19: Coronavirus disease 2019
E-Assessment: Electronic Assessment
E-learning platform: Electronic learning platform
IMS: ItemManagementSystem
MME: Master of Medical Education
OSCE: Objective Structured Clinical Examinations
SS 2020: Summer Semester 2020
UCAN: Umbrella Consortium for Assessment Networks
