# Supplementary material for: Impact of COVID-19 on digital medical education: compatibility of digital teaching and examinations with integrity and ethical principles
Source: Int J Educ Integr. 2021 Sep 7;17(1):18. doi: 10.1007/s40979-021-00084-8 (PMC8421088; doi:10.1007/s40979-021-00084-8)
Supplement: Supplementary file 1 — Additional file 1. [file 40979_2021_84_MOESM1_ESM.pdf]

Lehre und Prüfungen im Sommersemester 2020 in Corona-Zeiten

Sehr geehrte Damen und Herren,

liebe UCAN-Partner*innen,

vielen Dank für Ihre Bereitschaft an unserer Umfrage "Lehre und Prüfungen im Sommersemester 2020 in Corona Zeiten" teilzunehmen.

Hierfür bitten wir Sie den nachfolgenden Fragebogen auszufüllen, dessen Bearbeitung ca. 10 Minuten in Anspruch nehmen wird.

Selbstverständlich ist die Teilnahme an der Umfrage freiwillig und Ihre Daten werden ausschließlich anonymisiert zu Forschungszwecken weiterverwendet.

Mit freundlichen Grüßen,

Ihr UCAN-Team

Institut für Kommunikations- und Prüfungsforschung gGmbH
Wieblinger Weg 92a
69123 Heidelberg
www.cares.institute (http://www.cares.institute)

In dieser Umfrage sind 34 Fragen enthalten.

Allgemeine Daten

Welcher Institution gehören Sie an? *

Bitte geben Sie Ihre Antwort hier ein:

Welche Rolle als UCAN-Partner nehmen Sie ein (z.B. technischer Admin, Prüfungsbeauftragte*r, Lehrbeauftragte*r)? *

Bitte geben Sie Ihre Antwort hier ein:

In welchem Studiengang sind Sie als Dozierende*r bzw. Prüfende*r tätig?

Bitte wählen Sie eine der folgenden Antworten.

Bitte wählen Sie nur eine der folgenden Antworten aus:

- ◯ Medizin
- ◯ Zahnmedizin
- ◯ Veterinärmedizin
- ◯ Anderer Studiengang

In welchen Fachrichtungen sind Sie als Dozierende*r bzw. Prüfende*r tätig?

Bitte geben Sie Ihre Antwort hier ein:

Wie viele Studierende unterrichten Sie pro Semester insgesamt?

Bitte geben Sie die Anzahl in Zahlen wieder.

Bitte geben Sie Ihre Antwort hier ein:

Wie viele Studierende prüfen Sie pro Semester insgesamt?

Bitte geben die Sie Anzahl in Zahlen wieder.

Bitte geben Sie Ihre Antwort hier ein:

Lehre im Sommersemester 2020

Bitte beschreiben Sie inwiefern Ihre Lehre im Sommersemester 2020 durch dir Corona-Pandemie beeinflusst wurde (z.B. Ausfall von Lehrveranstaltungen, Umstellung von analogen zu digitalem Unterricht). Falls keine Einwirkungen auftraten, schreiben Sie bitte „Keine“.

Bitte geben Sie Ihre Antwort hier ein:

Welche (digitalen) Lehrformate kamen/kommen in Ihren Lehrveranstaltungen zum Einsatz?

Bitte wählen Sie alle zutreffenden Antworten aus.

	vor dem SS 2020	während dem SS 2020
**Es konnten keine Lehrveranstaltungen durchgeführt werden**	☐	☐
**Präsenzvorlesungen**	☐	☐
**Digitale synchrone Vorlesungen**	☐	☐
**Digitale asynchrone Vorlesungen (z.B. Podcasts)**	☐	☐
**Seminare mit Präsenzunterricht**	☐	☐
**Digitale synchrone Seminare**	☐	☐
**Kurse mit Präsenzunterricht**	☐	☐
**Digitale synchrone Kurse**	☐	☐
**Praktika mit Präsenz**	☐	☐
**Digitale Praktika**	☐	☐
**Skills-lab Trainings**	☐	☐

Folgende Lehrmethoden kamen/kommen im Sommersemester 2020 vermehrt zum Einsatz?

Bitte wählen Sie alle zutreffenden Antworten aus.

*

Beantworten Sie diese Frage nur, wenn folgende Bedingungen erfüllt sind:
Antwort war '' bei Frage '8 [B1]' (Welche (digitalen) Lehrformate kamen/kommen in Ihren Lehrveranstaltungen zum Einsatz? Bitte wählen Sie alle zutreffenden Antworten aus.)

Bitte wählen Sie die zutreffende Antwort für jeden Punkt aus:

	Ja	Nein
**Blended Learning**	◯	◯
**E-Learning**	◯	◯
**Inverted Classroom**	◯	◯

Sofern Sie interaktive Lehrveranstaltungen digital durchgeführt haben/durchführen, welche Videokonferenz-Systeme kamen/kommen hier zum Einsatz?

Bitte wählen Sie alle zutreffenden Antworten aus.

Beantworten Sie diese Frage nur, wenn folgende Bedingungen erfüllt sind:
Antwort war '' bei Frage '8 [B1]' (Welche (digitalen) Lehrformate kamen/kommen in Ihren Lehrveranstaltungen zum Einsatz? Bitte wählen Sie alle zutreffenden Antworten aus.)

	vor dem SS 2020	während dem SS 2020
**Adobe Connect**	☐	☐
**Cisco WebEx**	☐	☐
**Go to Meeting**	☐	☐
**Microsoft-Teams**	☐	☐
**Skype**	☐	☐
**Zoom**	☐	☐
**Elektronische Plattform, bereitgestellt von Ihrer Institution**	☐	☐
**Andere**	☐	☐
**Keine**	☐	☐

Welche der folgenden Lernplattformen stellte/stellt Ihre Institution Lehrbeauftragten und Studierenden zur Verfügung?

Bitte wählen Sie alle zutreffenden Antworten aus.

Beantworten Sie diese Frage nur, wenn folgende Bedingungen erfüllt sind:
Antwort war '' bei Frage '8 [B1]' (Welche (digitalen) Lehrformate kamen/kommen in Ihren Lehrveranstaltungen zum Einsatz? Bitte wählen Sie alle zutreffenden Antworten aus.)

	Moodle	ILIAS	Hauseigene Plattform	Keine	Andere *(Mehrfachnennungen möglich)*
**Vor dem SS 2020**					
**Während dem SS 2020**					

COVID-19 Einsätze durch Medizinstudierende (z.B. Unterstützung in Testzentren) wurden als Studienleistung anerkannt.

Bitte wählen Sie eine der folgenden Antworten.

Bitte wählen Sie nur eine der folgenden Antworten aus:

◯ Ja
◯ Nein

Falls Sie Fragen bezüglich "Lehre im Sommersemester 2020" durch die Vorgabe von Antworten nicht adäquat beantworten konnten, haben Sie hier Gelegenheit zusätzliche Anmerkungen zu machen.

Bitte geben Sie Ihre Antwort hier ein:

Prüfungen im Sommersemester 2020

Wurden/werden nach Ausbruch der Corona Pandemie im Sommersemester 2020 Prüfungen durchgeführt?

Bitte wählen Sie eine der folgenden Antworten.

*

Bitte wählen Sie nur eine der folgenden Antworten aus:

- ◯ Ja
- ◯ Nein

Wie viele Prüfungen inklusive Wiederholungsprüfungen konnten/können Sie im Sommersemester 2020 durchführen?

Bitte geben Sie die Anzahl in Zahlen wieder.

*

Beantworten Sie diese Frage nur, wenn folgende Bedingungen erfüllt sind:
Antwort war 'Ja' bei Frage '14 [C1]' (Wurden/werden nach Ausbruch der Corona Pandemie im Sommersemester 2020 Prüfungen durchgeführt? Bitte wählen Sie eine der folgenden Antworten.)

Bitte geben Sie Ihre Antwort hier ein:

Wie viele geplante Prüfungen inklusive Wiederholungsprüfungen konnten/können Sie im Sommersemester 2020 nicht durchführen?

Bitte geben Sie die Anzahl in Zahlen wieder.

*

Bitte geben Sie Ihre Antwort hier ein:

Prüfungen, die im Sommersemester 2020 nicht bestanden werden, werden an Ihrer Institution als Fehlversuch gewertet.

Bitte wählen Sie eine der folgenden Antworten.

Beantworten Sie diese Frage nur, wenn folgende Bedingungen erfüllt sind:
Antwort war 'Ja' bei Frage '14 [C1]' (Wurden/werden nach Ausbruch der Corona Pandemie im Sommersemester 2020 Prüfungen durchgeführt? Bitte wählen Sie eine der folgenden Antworten.)

Bitte wählen Sie nur eine der folgenden Antworten aus:

- ◯ Ja
- ◯ Nein

Wie wurden/werden bei Ihnen die schriftlichen Prüfungen hauptsächlich durchgeführt?

Bitte wählen Sie für jede Teilfrage eine Antwortmöglichkeit aus.

*

Beantworten Sie diese Frage nur, wenn folgende Bedingungen erfüllt sind:
Antwort war 'Ja' bei Frage '14 [C1]' (Wurden/werden nach Ausbruch der Corona Pandemie im Sommersemester 2020 Prüfungen durchgeführt? Bitte wählen Sie eine der folgenden Antworten.)

Bitte wählen Sie die zutreffende Antwort für jeden Punkt aus:

	**vor dem SS 2020**	**während dem SS 2020**
**Präsenzprüfung (an der Hochschule vor Ort)**	◯	◯
**Online-Prüfung (nicht vor Ort)**	◯	◯
**Es findet/fand keine schriftliche Prüfung statt**	◯	◯

Falls schriftliche Online-Prüfungen stattfanden/stattfinden, welche digitalen Tools/Software kommen/kamen zum Einsatz?

Bitte nennen Sie falls möglich Name und Hersteller.

Beantworten Sie diese Frage nur, wenn folgende Bedingungen erfüllt sind:
Antwort war 'Ja' bei Frage '14 [C1]' (Wurden/werden nach Ausbruch der Corona Pandemie im Sommersemester 2020 Prüfungen durchgeführt? Bitte wählen Sie eine der folgenden Antworten.) *und* Antwort war 'Online-Prüfung (nicht vor Ort)' bei Frage '18 [C5]' (Wie wurden/werden bei Ihnen die schriftlichen Prüfungen hauptsächlich durchgeführt? Bitte wählen Sie für jede Teilfrage eine Antwortmöglichkeit aus. (**während** dem SS 2020))

Bitte geben Sie Ihre Antwort hier ein:

Mit welchem Medium wurden/werden bei Ihnen schriftliche Prüfungen hauptsächlich durchgeführt?

Bitte wählen Sie für jede Teilfrage eine Antwortmöglichkeit aus.

*

Beantworten Sie diese Frage nur, wenn folgende Bedingungen erfüllt sind:
Antwort war 'Ja' bei Frage '14 [C1]' (Wurden/werden nach Ausbruch der Corona Pandemie im Sommersemester 2020 Prüfungen durchgeführt? Bitte wählen Sie eine der folgenden Antworten.) *und* Antwort war NICHT 'Es findet/fand keine schriftliche Prüfung statt' bei Frage '18 [C5]' (Wie wurden/werden bei Ihnen die schriftlichen Prüfungen hauptsächlich durchgeführt? Bitte wählen Sie für jede Teilfrage eine Antwortmöglichkeit aus. (**während** dem SS 2020))

Bitte wählen Sie die zutreffende Antwort für jeden Punkt aus:

	vor dem SS 2020	während dem SS 2020
**Tablet**	○	○
**Desktop**	○	○
**Papier**	○	○

Wie wurden/werden *mündliche Prüfungen* bei Ihnen hauptsächlich durchgeführt?

Bitte wählen Sie für jede Teilfrage eine Antwortmöglichkeit aus.

*

Beantworten Sie diese Frage nur, wenn folgende Bedingungen erfüllt sind:
Antwort war 'Ja' bei Frage '14 [C1]' (Wurden/werden nach Ausbruch der Corona Pandemie im Sommersemester 2020 Prüfungen durchgeführt? Bitte wählen Sie eine der folgenden Antworten.)

Bitte wählen Sie die zutreffende Antwort für jeden Punkt aus:

	vor dem SS 2020	während dem SS 2020
**Präsenzprüfung (an der Hochschule vor Ort)**	○	○
**Online-Prüfung (nicht vor Ort)**	○	○
**Es findet/fand keine mündlichen Prüfung statt**	○	○

Welche weiteren praktischen Prüfungsformate (z.B. DOPS, Mini CEX) wurden bei Ihnen genutzt?

Mehrfachnennungen sind möglich. Falls Sie keine weiteren praktischen Prüfungen durchgeführt haben, schreiben sie bitte "Keine".

*

Beantworten Sie diese Frage nur, wenn folgende Bedingungen erfüllt sind:
Antwort war 'Ja' bei Frage '14 [C1]' (Wurden/werden nach Ausbruch der Corona Pandemie im Sommersemester 2020 Prüfungen durchgeführt? Bitte wählen Sie eine der folgenden Antworten.)

Falls mündliche Online-Prüfungen stattfanden/stattfinden, welche digitalen Konferenzsysteme kommen/kamen zum Einsatz? *

Beantworten Sie diese Frage nur, wenn folgende Bedingungen erfüllt sind:
Antwort war 'Ja' bei Frage '14 [C1]' (Wurden/werden nach Ausbruch der Corona Pandemie im Sommersemester 2020 Prüfungen durchgeführt? Bitte wählen Sie eine der folgenden Antworten.) *und* Antwort war 'Online-Prüfung (nicht vor Ort)' bei Frage '22 [C8]' (Wie wurden/werden mündliche Prüfungen bei Ihnen hauptsächlich durchgeführt? Bitte wählen Sie für jede Teilfrage eine Antwortmöglichkeit aus. (während dem SS 2020))

Bitte geben Sie Ihre Antwort hier ein:

Welches Medium wurde/wird bei Ihnen hauptsächlich für die Bewertung der Studierendenleistung in *mündlichen Prüfungen* eingesetzt?

Bitte wählen Sie für jede Teilfrage eine Antwortmöglichkeit aus.

Beantworten Sie diese Frage nur, wenn folgende Bedingungen erfüllt sind:
Antwort war 'Ja' bei Frage '14 [C1]' (Wurden/werden nach Ausbruch der Corona Pandemie im Sommersemester 2020 Prüfungen durchgeführt? Bitte wählen Sie eine der folgenden Antworten.) *und* Antwort war NICHT 'Es findet/fand keine mündlichen Prüfung statt' bei Frage '22 [C8]' (Wie wurden/werden mündliche Prüfungen bei Ihnen hauptsächlich durchgeführt? Bitte wählen Sie für jede Teilfrage eine Antwortmöglichkeit aus. (während dem SS 2020))

Bitte wählen Sie die zutreffende Antwort für jeden Punkt aus:

	vor dem SS 2020	während dem SS 2020
**Tablet**	○	○
**Desktop**	○	○
**Papier**	○	○

Wie wurden/werden *OSCE-Prüfungen* bei Ihnen hauptsächlich durchgeführt?

Bitte wählen Sie für jede Teilfrage eine Antwortmöglichkeit aus.

*

Beantworten Sie diese Frage nur, wenn folgende Bedingungen erfüllt sind:
Antwort war 'Ja' bei Frage '14 [C1]' (Wurden/werden nach Ausbruch der Corona Pandemie im Sommersemester 2020 Prüfungen durchgeführt? Bitte wählen Sie eine der folgenden Antworten.)

Bitte wählen Sie die zutreffende Antwort für jeden Punkt aus:

	**vor dem SS 2020**	**während dem SS 2020**
**Präsenzprüfung (an der Hochschule vor Ort)**	◯	◯
**Online-Prüfung (nicht vor Ort)**	◯	◯
**Es findet/fand keine OSCE Prüfung statt**	◯	◯

Falls digitale online-basierte OSCE-Prüfungen stattfanden/stattfinden, welche digitalen Konferenzsysteme/Tools/Software kommen/kamen zum Einsatz?

Beantworten Sie diese Frage nur, wenn folgende Bedingungen erfüllt sind:
Antwort war 'Ja' bei Frage '14 [C1]' (Wurden/werden nach Ausbruch der Corona Pandemie im Sommersemester 2020 Prüfungen durchgeführt? Bitte wählen Sie eine der folgenden Antworten.) *und* Antwort war 'Online-Prüfung (nicht vor Ort)' bei Frage '25 [C11]' (Wie wurden/werden OSCE-Prüfungen bei Ihnen hauptsächlich durchgeführt? Bitte wählen Sie für jede Teilfrage eine Antwortmöglichkeit aus. (**während** dem SS 2020))

Bitte geben Sie Ihre Antwort hier ein:

Welches Medium wurde/wird bei Ihnen hauptsächlich für die Bewertung der Studierendenleistung in *OSCE Prüfungen* eingesetzt?

Bitte wählen Sie für jede Teilfrage eine Antwortmöglichkeit aus.

Beantworten Sie diese Frage nur, wenn folgende Bedingungen erfüllt sind:
Antwort war 'Ja' bei Frage '14 [C1]' (Wurden/werden nach Ausbruch der Corona Pandemie im Sommersemester 2020 Prüfungen durchgeführt? Bitte wählen Sie eine der folgenden Antworten.) *und* Antwort war NICHT 'Es findet/fand keine OSCE Prüfung statt' bei Frage '25 [C11]' (Wie wurden/werden OSCE-Prüfungen bei Ihnen hauptsächlich durchgeführt? Bitte wählen Sie für jede Teilfrage eine Antwortmöglichkeit aus. (**während** dem SS 2020))

Bitte wählen Sie die zutreffende Antwort für jeden Punkt aus:

	**vor dem SS 2020**	**während dem SS 2020**
**Tablet**	◯	◯
**Desktop**	◯	◯
**Papier**	◯	◯

Falls Sie Fragen bezüglich "Prüfungen im Sommersemester 2020" durch die Vorgabe von Antworten nicht adäquat beantworten konnten, haben Sie hier Gelegenheit zusätzliche Anmerkungen zu machen.

Bitte geben Sie Ihre Antwort hier ein:

Schätzen Sie als Lehr- bzw. Prüfungsbeauftragte*r die digitale Umsetzung von Lehre und Prüfung zukünftig als wichtig ein?

Bitte wählen Sie eine der folgenden Antworten.

Bitte wählen Sie nur eine der folgenden Antworten aus:

◯ Ja
◯ Nein

Welche Hindernisse traten im Sommersemester 2020 an Ihrer Institution auf?

Bitte geben Sie Ihre Antwort hier ein:

Glauben Sie, dass die Erfahrungen mit der digitalen Umsetzung von Lehre und Prüfung, die während der Pandemie gewonnen wurde, zukünftig von Nutzen sein wird?

Bitte wählen Sie eine der folgenden Antworten.

Bitte wählen Sie nur eine der folgenden Antworten aus:

◯ Ja
◯ Nein

Abschließende Gesamtbeurteilung

Könnte digitale Lehre Ihrer Meinung nach die Präsenzlehre ersetzen?

Bitte wählen Sie eine der folgenden Antworten.

Bitte wählen Sie nur eine der folgenden Antworten aus:

- ◯ Ja
- ◯ Nein
- ◯ Teilweise

Könnten digitale Prüfungen Ihrer Meinung nach die Präsenzprüfungen ersetzen?

Bitte wählen Sie eine der folgenden Antworten.

Bitte wählen Sie nur eine der folgenden Antworten aus:

- ◯ Ja
- ◯ Nein
- ◯ Teilweise

Vielleicht haben wir es versäumt, eine wichtige Frage zu stellen. Bitte fügen Sie bei Bedarf unten weitere Kommentare hinzu oder schreiben Sie "Keine".

Bitte geben Sie Ihre Antwort hier ein:

Das UCAN-Team bedankt sich herzlich für Ihre Teilnahme an unserer Umfrage!

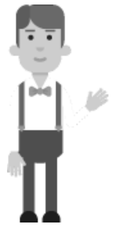

30.09.2020 – 23:55

Übermittlung Ihres ausgefüllten Fragebogens:
Vielen Dank für die Beantwortung des Fragebogens.
