# Supplementary material for: Impact of COVID-19 on digital medical education: compatibility of digital teaching and examinations with integrity and ethical principles
Source: Int J Educ Integr. 2021 Sep 7;17(1):18. doi: 10.1007/s40979-021-00084-8 (PMC8421088; doi:10.1007/s40979-021-00084-8)
Supplement: Supplementary file 2 — Additional file 2. [file 40979_2021_84_MOESM2_ESM.pdf]

Follow-up survey

Sehr geehrte Damen und Herren, liebe UCAN-Partner*innen,

vielen Dank für Ihre Bereitschaft an unserer Umfrage "Nachbefragung: Lehre und Prüfungen während der Corona-Pandemie" teilzunehmen. Hierfür bitten wir Sie den nachfolgenden Fragebogen auszufüllen, dessen Bearbeitung ca. 10 Minuten in Anspruch nehmen wird. Selbstverständlich ist die Teilnahme an der Umfrage freiwillig und Ihre Daten werden ausschließlich anonymisiert zu Forschungszwecken weiterverwendet.

Mit herzlichen Grüßen,

Ihr UCAN-Team

In dieser Umfrage sind 18 Fragen enthalten.

Technische Aspekte zu Lehre und Prüfungen

Im Rahmen unserer Erstbefragung wurde deutlich, dass es sowohl in Bezug auf die Lehre also auch auf die Prüfungen zu Hindernissen kam, da eine angemessene technische Ausstattung nicht zur Verfügung stand.

War dies an Ihrer Fakultät auch der Fall?

Bitte wählen Sie eine der folgenden Antworten:
Bitte wählen Sie nur eine der folgenden Antworten aus:

- ◯ Ja, an meiner Fakultät fehlte angemessene Ausstattung.
- ◯ Nein, meine Fakultät war gut ausgestattet.

Wie sieht die Situation bezüglich der technischen Ausstattung mittlerweile aus? Wurden ggf. nachhaltige Lösungen geschaffen?

Beantworten Sie diese Frage nur, wenn folgende Bedingungen erfüllt sind:
Antwort war 'Ja, an meiner Fakultät fehlte angemessene Ausstattung.' bei Frage '1 [A1]' (Im Rahmen unserer Erstbefragung wurde deutlich, dass es sowohl in Bezug auf die Lehre also auch auf die Prüfungen zu Hindernissen kam, da eine angemessene technische Ausstattung nicht zur Verfügung stand. War dies an Ihrer Fakultät auch der Fall?)

Bitte geben Sie Ihre Antwort hier ein:

Einige Umfrageteilnehmende gaben an, dass für jedes Fach andere Plattformen und/oder technische Lösungen bereitgestellt wurden. Gibt es diesbezüglich mittlerweile einheitliche (Infra)Strukturen?

Bitte geben Sie Ihre Antwort hier ein:

Digitalisierung von Lehre und Prüfungen

Viele Teilnehmende der Umfrage gaben an, dass es aufgrund

- der Umstellung auf digitale Lehre und/oder digitale Prüfungen
- sowie der Einhaltung von Corona-Maßnahmen bei Prüfungen (z.B. Verteilung der Prüflinge auf mehrere Räumlichkeiten)

zu personellen Engpässen kam.

War dies an Ihrer Fakultät auch der Fall?

Bitte wählen Sie nur eine der folgenden Antworten aus:

- ◯ Ja
- ◯ Nein

Wurden neue personelle Ressourcen geschaffen? Wenn ja, in welchen Bereichen wurden diese geschaffen (z.B. Support)?

Beantworten Sie diese Frage nur, wenn folgende Bedingungen erfüllt sind:
Antwort war 'Ja' bei Frage '4 [B1]' (Viele Teilnehmende der Umfrage gaben an, dass es aufgrund der Umstellung auf digitale Lehre und/oder digitale Prüfungen sowie der Einhaltung von Corona-Maßnahmen bei Prüfungen (z.B. Verteilung der Prüflinge auf mehrere Räumlichkeiten) zu personellen Engpässen kam. War dies an Ihrer Fakultät auch der Fall?)

Bitte geben Sie Ihre Antwort hier ein:

Im Rahmen unserer vorherigen Umfrage, gaben mehrere Teilnehmende an, dass

- Lehrbeauftragte nicht geneigt waren auf digitale Lehre umzusteigen
- Lehrbeauftragte über ein nicht ausreichendes Training bezüglich digitaler Lehre verfügten
- gute didaktische Konzepte vernachlässigt wurden

um die Digitalisierung schnell voranzutreiben (die technisch einfachste Lösung wurde angestrebt).

War dies an Ihrer Fakultät auch der Fall?

Bitte wählen Sie nur eine der folgenden Antworten aus:

- ◯ Ja
- ◯ Nein

Falls ja, könnten Sie diese Aspekte näher erläutern? Hat sich mittlerweile etwas geändert? Wurden ggf. Schulungen/Beratungen angeboten, um die digitalen Kompetenzen der Lehrenden zu fördern? Hat sich die Bereitschaft/das Verständnis der Lehrenden bezüglich digitaler Strategien verändert? Konnten gute digitale Konzepte entwickelt werden?

Beantworten Sie diese Frage nur, wenn folgende Bedingungen erfüllt sind:
Antwort war 'Ja' bei Frage '6 [B4]' (Im Rahmen unserer vorherigen Umfrage, gaben mehrere Teilnehmende an, dass Lehrbeauftragte nicht geneigt waren auf digitale Lehre umzusteigen Lehrbeauftragte über ein nicht ausreichendes Training bezüglich digitaler Lehre verfügten gute didaktische Konzepte vernachlässigt wurden um die Digitalisierung schnell voranzutreiben (die technisch einfachste Lösung wurde angestrebt). War dies an Ihrer Fakultät auch der Fall?)

Bitte geben Sie Ihre Antwort hier ein:

Gerade praktische Lehrformate ließen sich nur sehr schwer digital umsetzen. Können praktische Kurse etc. mittlerweile wieder angeboten werden (ggf. auch teil-digitalisiert)? Bitte beschreiben Sie, wie an Ihrer Einrichtung mit praktischen Lehrformaten umgegangen wurden.

Bitte geben Sie Ihre Antwort hier ein:

Prüfungen

Bereits vor Beginn der Corona-Pandemie wurden an vielen Fakultäten schriftliche Prüfungen bereits elektronisch durchgeführt. Diese fanden aber zumeist in extra dafür bereitgestellten Räumlichkeiten der Fakultät und nicht als Distanz-Online Prüfungen statt. Während der Corona-Pandemie musste abgewogen werden, ob ein hoher Arbeitsaufwand für Präsenzprüfungen (z.B. Anmietung großer Räumlichkeiten; splitten der Kohorte auf mehrere Räume) oder Online-Prüfungen, deren Nutzung zu Beginn noch nicht als rechtsicher galt, genutzt wird.

In unserer vorherigen Umfrage wurde mehrfach angegeben, dass sich die Umfrageteilnehmenden sicher seien, dass schriftliche Distanz-Online-Prüfungen in der nahen Zukunft nicht durchzusetzen wären.

Wurde die Studienordnung/Prüfungsordnung an Ihrer Einrichtung angepasst um Heim-Online-Prüfungen zu ermöglichen?

Bitte wählen Sie nur eine der folgenden Antworten aus:

- ◯ Ja
- ◯ Nein

Kommen an Ihrer Fakultät summative Heim-Online Prüfungen zum Einsatz?

Definition: Summative Heim-Online-Prüfungen sind benotete, elektronische Prüfungen, welche in den eigenen Räumlichkeiten der Studierenden stattfinden.

Bitte wählen Sie nur eine der folgenden Antworten aus:

- ◯ Ja
- ◯ Nein

Welches Prüfungswerkzeug wird hierfür verwendet?

Beantworten Sie diese Frage nur, wenn folgende Bedingungen erfüllt sind:
Antwort war 'Ja' bei Frage '10 [C2]' (Kommen an Ihrer Fakultät summative Heim-Online Prüfungen zum Einsatz? Definition: Summative Heim-Online-Prüfungen sind benotete, elektronische Prüfungen, welche in den eigenen Räumlichkeiten der Studierenden stattfinden.)

Bitte geben Sie Ihre Antwort hier ein:

Wie viele Studierende konnten bisher in Heim-Online-Prüfungen geprüft werden?

Bitte geben Sie eine Zahl oder einen Zahlenbereich an.

Beantworten Sie diese Frage nur, wenn folgende Bedingungen erfüllt sind:
Antwort war 'Ja' bei Frage '10 [C2]' (Kommen an Ihrer Fakultät summative Heim-Online Prüfungen zum Einsatz? Definition: Summative Heim-Online-Prüfungen sind benotete, elektronische Prüfungen, welche in den eigenen Räumlichkeiten der Studierenden stattfinden.)

Bitte geben Sie Ihre Antwort hier ein:

Wenn Sie die Durchschnittsnoten von Online-Prüfungen und Präsenzprüfungen vergleichen, sind diese vergleichbar oder gibt es Differenzen? Sind Online-Prüfungen ggf. leichter oder schwerer als Präsenzprüfungen?

Beantworten Sie diese Frage nur, wenn folgende Bedingungen erfüllt sind:
Antwort war 'Ja' bei Frage '10 [C2]' (Kommen an Ihrer Fakultät summative Heim-Online Prüfungen zum Einsatz? Definition: Summative Heim-Online-Prüfungen sind benotete, elektronische Prüfungen, welche in den eigenen Räumlichkeiten der Studierenden stattfinden.)

Bitte geben Sie Ihre Antwort hier ein:

Wird eine Proctoring Software eingesetzt?

Proctoring: Überwachung der Studierenden mittels spezieller Software

Beantworten Sie diese Frage nur, wenn folgende Bedingungen erfüllt sind:
Antwort war 'Ja' bei Frage '10 [C2]' (Kommen an Ihrer Fakultät summative Heim-Online Prüfungen zum Einsatz? Definition: Summative Heim-Online-Prüfungen sind benotete, elektronische Prüfungen, welche in den eigenen Räumlichkeiten der Studierenden stattfinden.)

Bitte wählen Sie nur eine der folgenden Antworten aus:

- ◯ Ja
- ◯ Nein

Sind an Ihrer Fakultät Betrugsversuche oder Regelverstöße in Online Prüfungen bekannt geworden? Falls ja, worum handelte es sich dabei?

Beantworten Sie diese Frage nur, wenn folgende Bedingungen erfüllt sind:
Antwort war 'Ja' bei Frage '10 [C2]' (Kommen an Ihrer Fakultät summative Heim-Online Prüfungen zum Einsatz? Definition: Summative Heim-Online-Prüfungen sind benotete, elektronische Prüfungen, welche in den eigenen Räumlichkeiten der Studierenden stattfinden.)

Bitte geben Sie Ihre Antwort hier ein:

Eine relativ sichere Online-Prüfung kann nicht nur durch Proctoring, Blacklists zeitgleich laufender Programme etc. erzielt werden, sondern auch durch eine Anpassung der Prüfungs- bzw. Fragenformate. Hat eine solche Anpassung stattgefunden oder ist diese zukünftig angestrebt?

Proctoring: Überwachung der Studierenden mittels spezieller Software.

Blacklist: Liste an Programmen, deren Nutzung einen Start der Prüfung verhindert.

Beantworten Sie diese Frage nur, wenn folgende Bedingungen erfüllt sind:
Antwort war 'Ja' bei Frage '10 [C2]' (Kommen an Ihrer Fakultät summative Heim-Online Prüfungen zum Einsatz? Definition: Summative Heim-Online-Prüfungen sind benotete, elektronische Prüfungen, welche in den eigenen Räumlichkeiten der Studierenden stattfinden.)

Bitte geben Sie Ihre Antwort hier ein:

Wie wurden Online-Prüfungen bei den Studierenden aufgenommen. Kam es zu Beschwerden bezüglich der Chancengleichheit? Falls ja, worum handelte es sich hierbei?

Beantworten Sie diese Frage nur, wenn folgende Bedingungen erfüllt sind:
Antwort war 'Ja' bei Frage '10 [C2]' (Kommen an Ihrer Fakultät summative Heim-Online Prüfungen zum Einsatz? Definition: Summative Heim-Online-Prüfungen sind benotete, elektronische Prüfungen, welche in den eigenen Räumlichkeiten der Studierenden stattfinden.)

Bitte geben Sie Ihre Antwort hier ein:

Sollen Heim-Online-Prüfungen auch zukünftig eingesetzt werden, wenn Präsenzprüfungen wieder ohne Hygienekonzept durchgeführt werden dürfen?

Beantworten Sie diese Frage nur, wenn folgende Bedingungen erfüllt sind:
Antwort war 'Ja' bei Frage '10 [C2]' (Kommen an Ihrer Fakultät summative Heim-Online Prüfungen zum Einsatz? Definition: Summative Heim-Online-Prüfungen sind benotete, elektronische Prüfungen, welche in den eigenen Räumlichkeiten der Studierenden stattfinden.)

Bitte wählen Sie nur eine der folgenden Antworten aus:

- ◯ Ja
- ◯ Nein

Das UCAN-Team bedankt sich herzlich für Ihre Teilnahme an unserer Umfrage!

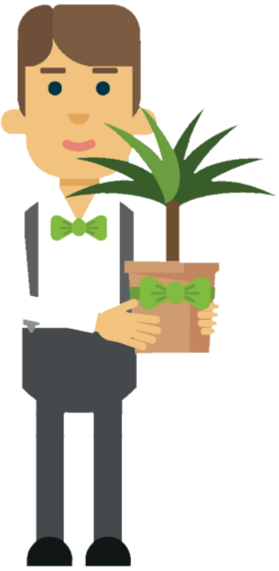

16.06.2021 – 23:59

Übermittlung Ihres ausgefüllten Fragebogens:
Vielen Dank für die Beantwortung des Fragebogens.
